# C-reactive Protein (CRP) Levels as a Predictor of Adverse Cardiovascular Events in Acute Myocardial Infarction: A Prospective Study

**DOI:** 10.7759/cureus.93943

**Published:** 2025-10-06

**Authors:** Muhammad Ahtesham, Sher Wali Khan, Sofia R Khan, Ayesha Fayyaz, Zarak Khan, Ikram Ullah, Yasir Ahmad

**Affiliations:** 1 Cardiology, Lady Reading Hospital - Medical Teaching Institute, Peshawar, PAK; 2 Adult Cardiology, Lady Reading Hospital - Medical Teaching Institute, Peshawar, PAK; 3 Internal Medicine, SUNY (State University of New York) Downstate Medical Center - SUNY Downstate Health Sciences University, Brooklyn, USA; 4 Cardiology, Peshawar Institute of Cardiology - Medical Teaching Institute, Peshawar, PAK; 5 Cardiology, Northwest General Hospital and Research Center, Peshawar, PAK; 6 Medical Science, Khyber Medical University Institute of Medical Sciences (KMU-IMS), Kohat, PAK

**Keywords:** acute myocardial infarction, cardiovascular outcomes, crp, high sensitivity crp, inflammation, major adverse cardiovascular events, mortality, prognostic marker, risk stratification

## Abstract

Introduction

Acute myocardial infarction (AMI) is a major cause of illness and mortality around the world. Anti-inflammatory biomarkers, especially C-reactive protein (CRP), which indicate the degree of cardiac injury and systemic inflammation, have been proposed as predictors of poor cardiovascular outcomes.

Objective

The main objective of this study is to evaluate the role of CRP levels at admission as a predictor of 30-day major adverse cardiovascular events (MACE) in patients with AMI.

Methodology

This prospective observational cohort study was conducted at Lady Reading Hospital and Medical Teaching Institute, Peshawar, Pakistan, over a 12-month period. A total of 200 patients presenting with confirmed AMI were enrolled. Baseline demographics, clinical characteristics, and laboratory parameters, including serum CRP, were recorded. Patients were stratified into three CRP groups: <5 mg/L, 5-10 mg/L, and >10 mg/L. The selected thresholds were based on established clinical interpretations of inflammatory activity, where CRP levels <5 mg/L indicate minimal inflammation, 5-10 mg/L reflect moderate inflammation, and >10 mg/L represent marked systemic inflammation, typically associated with acute disease states. The primary outcome was 30-day MACE, including death, reinfarction, and heart failure. Statistical analyses included Chi-square tests, one-way analysis of variance (ANOVA), and multivariable logistic regression to adjust for potential confounders.

Results

The mean age was 57.8 ± 10.6 years, with 132 (66%) males. Elevated CRP levels (>10 mg/L) were significantly associated with a higher incidence of MACE (19, 42.2%) compared to the moderate (21, 24.7%) and low CRP groups (6, 8.6%) (p < 0.001). Multivariable logistic regression confirmed CRP as an independent predictor of 30-day MACE (adjusted OR: 1.18 per 1 mg/L increase, 95% CI: 1.09-1.27, p < 0.001), alongside age, troponin, and time to hospital admission.

Conclusion

Admission CRP levels are a strong independent predictor of short-term adverse outcomes in AMI patients, and can be used for early risk stratification and management decisions.

## Introduction

Acute myocardial infarction (AMI) continues to rank among the world's top causes of morbidity and mortality, despite notable advancements in therapeutic approaches, diagnostic techniques, and preventive measures [1]. Atherosclerotic plaque rupture and subsequent thrombus formation, resulting in partial or total coronary artery occlusion, are the main pathophysiological factors of AMI [2]. Early diagnosis and prompt management of acute coronary syndrome (ACS) remain the cornerstone for preventing myocardial necrosis and improving clinical outcomes [3]. Traditionally, risk stratification in ACS has relied on clinical scores, electrocardiographic findings, and cardiac biomarkers, such as troponins [4]. However, growing evidence indicates that systemic inflammation plays a central role in the pathogenesis and complications of atherosclerotic cardiovascular disease, emphasizing the importance of identifying inflammatory biomarkers that can predict adverse outcomes [5].

C-reactive protein (CRP), a hepatic acute-phase reactant synthesized in response to interleukin-6 (IL-6), has emerged as one of the most widely studied indicators of systemic inflammation [6]. High-sensitivity CRP (hs-CRP) assays enable the detection of low CRP concentrations, making them valuable for assessing cardiovascular risk, even in apparently healthy individuals [7]. AMI development is strongly influenced by endothelial dysfunction (ED), plaque instability, and prothrombotic conditions, all of which are closely linked to elevated CRP levels [8]. Recent evidence also indicates a rising burden of cardiovascular and metabolic risk factors among younger populations, including metabolic syndrome, autoimmune conditions, and substance use, contributing to early-onset heart failure and ischemic heart disease [9]. ED, characterized by impaired vasodilation, inflammation, and thrombosis, remains a key mechanism connecting chronic kidney disease and cardiovascular morbidity, and continues to influence cardiovascular outcomes despite partial restoration following kidney transplantation [10]. Furthermore, raised baseline hs-CRP has been shown to independently predict major adverse cardiac or cerebrovascular events (MACE) at 24 months in patients with nonvalvular atrial fibrillation, underscoring the prognostic value of systemic inflammation in cardiovascular disease [11].

Several studies have demonstrated that CRP levels rise rapidly after myocardial necrosis and remain elevated for several days, suggesting its utility not only in predicting AMI development, but also in forecasting recurrent infarction, heart failure, arrhythmias, and mortality [11]. Despite the widespread use of CRP as an inflammatory marker, its prognostic significance in the specific context of AMI remains controversial [12]. While some studies have linked elevated CRP to poor short- and long-term outcomes, others have failed to demonstrate its independent predictive value after adjusting for traditional risk factors [13]. Moreover, data from low- and middle-income countries, including Pakistan, remain scarce, with most evidence derived from Western populations, where demographic, genetic, and environmental determinants differ substantially [14]. This underscores the need to evaluate CRP as a prognostic tool in diverse patient populations, where cardiovascular disease burden and healthcare resources present unique challenges [15,16].

Given the paucity of local evidence examining CRP as an independent predictor of adverse cardiovascular outcomes following AMI in the Pakistani population, this study aims to assess the predictive value of CRP levels for adverse cardiovascular events in patients presenting with AMI.

## Materials and methods

Study design and setting

This was a prospective observational cohort study conducted in accordance with the STROBE (Strengthening the Reporting of Observational Studies in Epidemiology) guidelines to ensure methodological transparency and comprehensive reporting. The study was carried out at Lady Reading Hospital - Medical Teaching Institute (LRH), Peshawar, Pakistan, over a 12-month period, from January 1, 2024, to December 31, 2024.

A prospective cohort design was selected to determine the prognostic relevance of serum CRP levels in routine clinical practice and real-world conditions. The study setting included the Emergency Department and Cardiology Unit of LRH, a tertiary care center serving a large urban and semi-urban population. The primary objective was to assess the predictive impact of baseline serum CRP levels on adverse cardiovascular outcomes in patients presenting with AMI.

Study population

All patients with a verified diagnosis of AMI who were at least 18 years old were eligible for enrollment. Patients with chronic inflammatory conditions, active infections, malignancy under current therapy, recent major trauma or surgery within the past month, or systemic corticosteroid or immunosuppressant use were excluded. AMI diagnosis followed World Health Organization (WHO) criteria, which include typical ACS symptoms (chest pain lasting longer than 20 minutes), electrocardiographic changes indicative of myocardial infarction, and elevated cardiac biomarkers, preferably troponin I or T. Patients who refused to give consent were also excluded.

Sample size and calculation

The WHO's single-proportion formula was used to determine the sample size $n = \frac{Z^2 \times p \times (1 - p)}{d^2}$ [17]. Here, Z = 1.96 (for 95% confidence), p = 0.13 (anticipated 30-day incidence of composite MACE, based on an open-access AMI/STEMI (ST-elevation myocardial infarction) cohort from a similar South Asian population [18]), and d = 0.05 (desired margin of error). Using this formula, the minimum sample size was calculated as 178. The sample size was increased by 10% to account for potential attrition or missing data, resulting in a target of approximately 196 cases. For operational convenience and to ensure an adequate number of outcome events for stable multivariable analyses and subgroup evaluations, a conservative recruitment cap of 200 participants was set. This approach balances precision with feasibility and supports adjustment for multiple confounding variables in logistic regression models.

Data collection procedures

Each participant gave informed consent following AMI stabilization and confirmation. Baseline demographic information collected included age, sex, and cardiovascular risk factors, such as diabetes, hypertension, hyperlipidemia, smoking, and a family history of ischemic heart disease. Blood pressure, heart rate, and the interval between the onset of symptoms and hospital presentation were documented during admission. Laboratory tests performed included CRP levels, cardiac troponins, complete blood count, renal function tests, lipid profile, and fasting glucose.

CRP sampling and analysis

Blood samples for CRP were collected at admission, prior to any major interventions when possible, and analyzed using an automated immunoturbidimetric assay in the hospital laboratory according to standard operating procedures. The average time from symptom onset to CRP sample collection was 5.8 ± 2.4 hours. In addition, serum CRP was re-evaluated at the 30-day follow-up visit in all surviving participants to assess the persistence or resolution of the inflammatory response. Follow-up samples were analyzed using the same automated immunoturbidimetric assay and laboratory protocol to ensure consistency of measurements.

Clinical management documentation

Management details, including the type of reperfusion strategy (primary percutaneous coronary intervention (PCI), thrombolysis, or conservative medical management) and the use of guideline-directed medications, were also documented.

Follow-up and outcome assessment

Patients were followed during hospitalization for adverse cardiovascular events and subsequently at 30 days post-admission through clinic visits or structured telephone interviews. All-cause mortality, recurrent myocardial infarction, and new or worsening heart failure necessitating hospitalization were the primary outcomes. Additionally, 30-day re-hospitalization and hospital stay length were secondary outcomes.

Statistical analysis

Statistical analysis was performed using IBM SPSS Statistics for Windows, Version 26 (Released 2018; IBM Corp., Armonk, NY, USA). The mean ± SD or median and interquartile range, as well as the frequency and percentages of categorical variables, were used to report continuous data. Analysis of variance (ANOVA) or t-tests were used to compare groups stratified based on CRP levels, and Chi-square tests were used to compare categorical variables. The relationship between baseline CRP and 30-day adverse cardiovascular events was determined using logistic regression. Multivariable logistic regression was used to control for potential confounders, including age, sex, smoking status, diabetes, hypertension, time from symptom onset to hospital admission, troponin levels, renal function, and reperfusion technique. Regarding adjusted ORs, 95% CIs were reported. p-values less than 0.05 were regarded as statistically significant. Sensitivity analysis involved investigating various cutoff points for classification and treating CRP as a continuous variable.

Ethical considerations

The study protocol was reviewed and approved by LRH Peshawar's Institutional Review Board (IRB) (Approval No. 941/LRH/MTI; dated December 13, 2023). Informed consent was obtained in writing from each participant or their legal guardian. By de-identifying data and storing it securely in password-protected folders accessible only to research investigators, patient confidentiality was preserved.

## Results

During the study period, 200 individuals with AMI were included. As summarized in Table 1, the study population had a mean age of approximately 58 years, with a predominance of male participants. Hypertension, diabetes mellitus, and smoking were the most frequently observed comorbidities and risk factors. Across the CRP strata, patients with higher CRP levels tended to be slightly older and exhibited a greater prevalence of hypertension, diabetes, and smoking history, indicating a consistent pattern of elevated inflammatory burden among those with multiple cardiovascular risk factors. The baseline characteristics were balanced, as evidenced by the lack of statistically significant differences between the CRP groups in age, sex, hypertension, diabetes, smoking, and family history (p > 0.05).

**Table 1:** Baseline Demographics and Clinical Characteristics of Patients (n = 200) Continuous variables were presented as mean ± SD, and categorical variables as n (%). For group comparisons, ANOVA and Chi-square tests were employed. No statistically significant differences were observed between the CRP groups (p > 0.05). ANOVA, Analysis of Variance; CRP, C-reactive Protein

Variable	Total (n = 200)	CRP <5 mg/L (n = 70)	CRP 5-10 mg/L (n = 85)	CRP >10 mg/L (n = 45)	p-value (test)	Test	Test value
Age, mean ± SD	57.8 ± 10.6	56.2 ± 9.8	58.5 ± 11.2	60.1 ± 10.8	0.08	ANOVA	F = 2.56
Male, n (%)	132 (66)	46 (65.7)	57 (67.1)	29 (64.4)	0.95	Chi-square	χ² = 0.10
Hypertension, n (%)	110 (55)	32 (45.7)	52 (61.2)	26 (57.8)	0.12	Chi-square	χ² = 4.19
Diabetes, n (%)	82 (41)	24 (34.3)	37 (43.5)	21 (46.7)	0.30	Chi-square	χ² = 2.39
Smoking, n (%)	94 (47)	28 (40)	42 (49.4)	24 (53.3)	0.28	Chi-square	χ² = 2.55
Family history, n (%)	38 (19)	10 (14.3)	18 (21.2)	10 (22.2)	0.43	Chi-square	χ² = 1.68

The baseline laboratory values of patients stratified by admission CRP levels are shown in Table 2. Across CRP strata, patients with higher CRP levels tended to be slightly older and exhibited a greater prevalence of hypertension, diabetes, and smoking history, suggesting a higher inflammatory and cardiovascular risk burden. The mean CRP level for the total cohort was 7.4 ± 3.2 mg/L, with subgroup means of 3.6 ± 0.8 mg/L in the low CRP group, 7.1 ± 1.5 mg/L in the intermediate group, and 12.3 ± 2.1 mg/L in the high CRP group (p < 0.001). Notably, patients in the high CRP group demonstrated markedly elevated median troponin I levels (16.8 ng/mL, IQR 12.4-22.5) compared to the low CRP group (10.1 ng/mL, IQR 7.2-13.8; p = 0.002), indicating greater myocardial injury. Although mean serum creatinine was slightly higher in the high CRP group (1.2 ± 0.4 mg/dL) than in the low CRP group (1.0 ± 0.2 mg/dL), this difference did not reach statistical significance (p = 0.05). These results suggest that higher troponin levels, and possibly higher renal stress, are linked to elevated CRP levels at admission. Serial CRP measurements demonstrated a significant decline from admission to 30 days (mean 7.4 ± 3.2 vs. 3.1 ± 1.6 mg/L, p < 0.001), consistent with the expected resolution of the acute inflammatory phase following AMI. Although CRP levels normalized in most patients, those with persistently elevated values (>5 mg/L at 30 days) were more likely to experience adverse cardiovascular outcomes. These follow-up CRP values are summarized in Table 2.

**Table 2:** Laboratory Parameters by CRP Levels on Admission (n = 200) The median (IQR) represents non-normal data, while the mean ± SD represents normally distributed data. CRP groups were compared using ANOVA. Admission CRP showed significant differences across strata (p < 0.001), whereas follow-up CRP at 30 days showed a marked decline across all groups, without significant intergroup variation (p = 0.09). Troponin and admission CRP revealed significant differences (p < 0.05), while creatinine displayed a non-significant trend (p = 0.05). ANOVA, Analysis of Variance; CRP, C-reactive Protein; IQR, Interquartile Range

Variable	Total (n = 200)	CRP <5 mg/L	CRP 5-10 mg/L	CRP >10 mg/L	p-value (test)	Test	Test values
CRP, mg/L (admission)	7.4 ± 3.2	3.6 ± 0.8	7.1 ± 1.5	12.3 ± 2.1	<0.001	ANOVA	F = 115.4
CRP, mg/L (30-day follow-up)	3.1 ± 1.6	2.8 ± 1.2	3.2 ± 1.5	3.6 ± 1.8	0.09	ANOVA	F = 2.42
Troponin I, ng/mL, median (IQR)	12.6 (8.2-18.5)	10.1 (7.2-13.8)	12.5 (8.5-17.8)	16.8 (12.4-22.5)	0.002	ANOVA	F = 6.45
Creatinine, mg/dL, mean ± SD	1.1 ± 0.3	1.0 ± 0.2	1.1 ± 0.3	1.2 ± 0.4	0.05	ANOVA	F = 3.02

Table 3 summarizes the 30-day MACE stratified by CRP levels on admission. Overall, 46 (23%) patients experienced composite MACE within 30 days. The incidence was highest among patients with CRP >10 mg/L, affecting 19 (42.2%) patients, followed by 21 (24.7%) in the CRP 5-10 mg/L group and six (8.6%) in the CRP <5 mg/L group (p < 0.001). Regarding mortality, 18 (9%) patients died, with the high CRP group having far higher rates (8, 17.8%) than the moderate CRP group (8, 9.4%) and the low CRP group (2, 2.9%) (p = 0.02). In terms of reinfarction, 12 (6%) patients experienced this event, which was more common in those with higher CRP levels: five (11.1%) in the high CRP group, six (7.1%) in the moderate group, and one (1.4%) in the low group (p = 0.04). A numerical rise in heart failure was associated with higher CRP levels - six (13.3%) in the high CRP group, seven (8.2%) in the moderate group, and three (4.3%) in the low group - affecting 16 (8%) of the patients overall, although this difference was not statistically significant (p = 0.10).

**Table 3:** Thirty-Day MACE by CRP Level (n = 200) Data are presented as number (n) and percentage (%). Comparisons across baseline and follow-up CRP categories were performed using the Chi-square test. Higher baseline and persistently elevated CRP levels (>10 mg/L) were significantly associated with increased composite MACE (p < 0.001), death (p = 0.02), and reinfarction (p = 0.04). Follow-up CRP was reassessed at 30 days, and persistent elevation correlated with adverse outcomes. MACE, Major Adverse Cardiovascular Events; CRP, C-reactive Protein

Event	Total, n	Total, %	Baseline CRP <5 mg/L, n (%)	Baseline CRP 5-10 mg/L, n (%)	Baseline CRP >10 mg/L, n (%)	Follow-up CRP <5 mg/L, n (%)	Follow-up CRP 5-10 mg/L, n (%)	Follow-up CRP >10 mg/L, n (%)	p-value (test)	Test	Test value
MACE	46	23	6 (8.6)	21 (24.7)	19 (42.2)	3 (5.1)	14 (21.2)	29 (43.9)	<0.001	χ²	18.76
Death	18	9	2 (2.9)	8 (9.4)	8 (17.8)	1 (1.7)	4 (6.1)	13 (19.7)	0.02	χ²	7.61
Reinfarction	12	6	1 (1.4)	6 (7.1)	5 (11.1)	0 (0.0)	3 (4.5)	9 (13.6)	0.04	χ²	6.36
Heart failure	16	8	3 (4.3)	7 (8.2)	6 (13.3)	2 (3.0)	5 (7.6)	9 (13.6)	0.10	χ²	4.52

**Figure 1:**
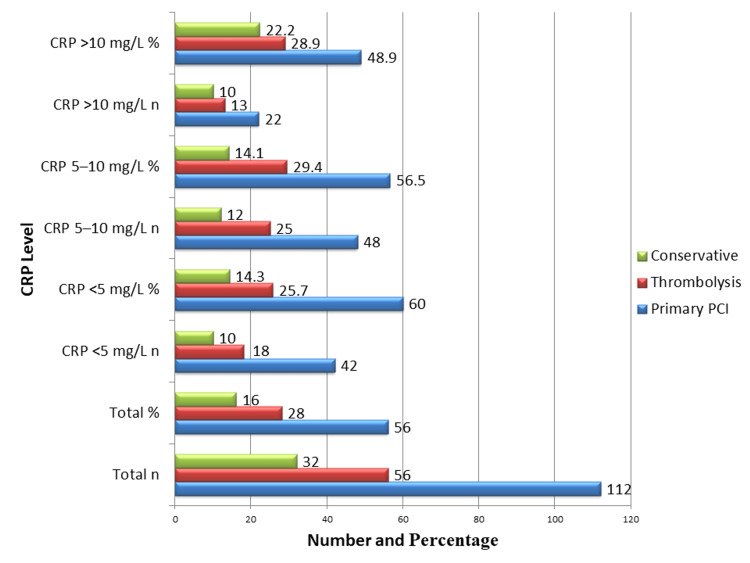
Type of Management by CRP Levels (n = 200) Data are presented as n (%); the Chi-square test was used. No significant differences were observed in management strategies: conservative (p = 0.42), thrombolysis (p = 0.88), and PCI (p = 0.49). CRP, C-reactive Protein; PCI, Percutaneous Coronary Intervention

Figure 1 illustrates the management strategies employed for patients across different CRP levels on admission. Overall, 112 (56%) patients underwent primary PCI, 56 (28%) received thrombolysis, and 32 (16%) were managed conservatively. Among patients with CRP <5 mg/L, 42 (60%) underwent primary PCI, 18 (25.7%) received thrombolysis, and 10 (14.3%) were managed conservatively. In the CRP 5-10 mg/L group, 48 (56.5%) had primary PCI, 25 (29.4%) received thrombolysis, and 12 (14.1%) were managed conservatively. In the CRP >10 mg/L group, 22 (48.9%) underwent primary PCI, 13 (28.9%) received thrombolysis, and 10 (22.2%) were managed conservatively. The choice of initial clinical therapy was not influenced by CRP levels, as evidenced by the lack of statistically significant variations in treatment approaches between CRP groups (p > 0.05). This suggests that baseline CRP was independent of the treatment modality applied.

**Figure 2:**
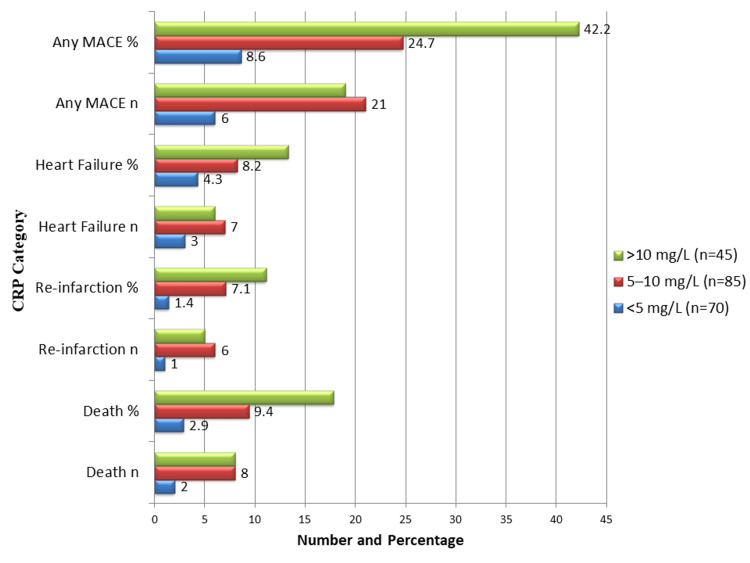
CRP Levels and Individual MACE Components (n = 200) Data are shown as n (%); Chi-square test used. Higher CRP levels (>10 mg/L) were significantly associated with death (p = 0.02), reinfarction (p = 0.04), and MACE (p < 0.001), while the association with heart failure was not statistically significant (p = 0.10). CRP, C-reactive Protein; MACE, Major Adverse Cardiovascular Events

Figure 2 illustrates the association between the 30-day MACE constituent components and CRP levels at admission. Patients with CRP >10 mg/L had the highest mortality, affecting eight (17.8%) patients, and reinfarction occurred in five (11.1%) patients. In comparison, the moderate CRP group (5-10 mg/L) had eight (9.4%) deaths and six (7.1%) reinfarctions, while the low CRP group (<5 mg/L) had two (2.9%) deaths and one (1.4%) reinfarction. Heart failure incidence increased with higher CRP levels - six (13.3%) in the high CRP group, seven (8.2%) in the moderate group, and three (4.3%) in the low group - but this difference was not statistically significant (p = 0.10). Composite MACE was highest in the high CRP group, affecting 19 (42.2%) patients, followed by 21 (24.7%) in the moderate group and six (8.6%) in the low CRP group (p < 0.001). These results support the use of elevated CRP levels as a reliable biomarker for risk stratification, demonstrating their strong predictive value for short-term major cardiovascular events in patients with AMI at the time of admission.

The multivariable logistic regression analysis for 30-day MACE predictors in AMI patients is shown in Table 4. An 18% increased risk of MACE was independently associated with every 1 mg/L increase in CRP (adjusted OR: 1.18; 95% CI: 1.09-1.27; p < 0.001). Additionally, higher troponin levels (adjusted OR: 1.08 per 1 ng/mL; 95% CI: 1.03-1.12; p = 0.001), older age (adjusted OR: 1.04 per year; 95% CI: 1.01-1.07; p = 0.01), and a longer time between symptom onset and hospital admission (adjusted OR: 1.06 per hour; 95% CI: 1.01-1.12; p = 0.02) also independently increased the risk. Male sex, diabetes, hypertension, smoking, and reperfusion strategy (PCI vs. conservative) did not show significant independent associations with 30-day MACE (all p > 0.05). These findings confirm that baseline CRP, age, troponin, and time to hospital arrival are the most critical independent predictors of short-term cardiovascular outcomes in AMI patients.

**Table 4:** Multivariable Logistic Regression for 30-Day MACE (n = 200) * denotes p < 0.05. Crude (unadjusted) and adjusted ORs (95% CIs) are displayed; multivariable logistic regression was employed. The 30-day MACE was significantly predicted by CRP (p < 0.001), age (p = 0.01), troponin (p = 0.001), and time to hospital admission (p = 0.02), but not by sex, diabetes, hypertension, smoking, or reperfusion strategy (all p > 0.05). CRP, C-reactive Protein; MACE, Major Adverse Cardiovascular Events; PCI, Percutaneous Coronary Intervention

Variable	Crude OR	95% CI	p-value	Adjusted OR	95% CI	p-value
CRP (per 1 mg/L increase)	1.21	1.12-1.30	<0.001*	1.18	1.09-1.27	<0.001*
Age (per year)	1.05	1.02-1.08	0.004*	1.04	1.01-1.07	0.01*
Male sex	1.15	0.65-2.05	0.63	1.12	0.62-2.02	0.70
Diabetes	1.55	0.88-2.73	0.13	1.45	0.82-2.56	0.20
Hypertension	1.36	0.79-2.32	0.27	1.31	0.75-2.30	0.35
Smoking	1.30	0.76-2.23	0.33	1.22	0.70-2.11	0.47
Time to hospital (per hour)	1.07	1.02-1.13	0.01*	1.06	1.01-1.12	0.02*
Troponin I (per 1 ng/mL)	1.10	1.05-1.15	<0.001*	1.08	1.03-1.12	0.001*
Reperfusion (PCI vs. conservative)	0.61	0.33-1.15	0.13	0.58	0.30-1.12	0.11

## Discussion

This study aimed to evaluate the predictive utility of CRP levels in forecasting short-term outcomes for patients with AMI. Our results indicate that elevated CRP levels upon admission are correlated with a higher risk of significant adverse cardiovascular outcomes - including death, reinfarction, and heart failure - within 30 days following an AMI. Specifically, the greatest risk of events was observed in patients with CRP levels greater than 10 mg/L. These findings highlight the importance of CRP as an independent indicator of poor outcomes in AMI patients.

It is well known that patients with ACS have a worse prognosis when their CRP level is high [19]. Regardless of other risk factors, higher CRP levels have been linked to an increased risk of death and recurrent cardiovascular events [20]. Additionally, research has shown that CRP levels measured during the acute stage of AMI correlate with infarct size and myocardial damage, indicating that CRP reflects the extent of myocardial injury [21]. Moreover, high CRP levels have been associated not only with a worse short-term prognosis after AMI, but also with the underlying role of inflammation in the pathophysiology of AMI [22].

These studies are consistent with our findings, which support the idea that CRP is a useful biomarker in the risk stratification of AMI patients [23]. The results, showing a correlation between increased CRP and an elevated incidence of MACE, are in line with the idea that inflammation is a decisive factor in AMI progression and outcomes [24]. Additionally, the independent predictive value of CRP, as revealed by our multivariate logistic regression analysis, aligns with past studies on the use of CRP alongside conventional risk factors [25].

Furthermore, our findings are similar to previous research conducted on South Asian populations, who have found the association between high CRP and cardiovascular events in the short term to be significant [15]. The same patterns were followed in European cohorts, with higher CRP levels being associated with larger infarct sizes and 30-day mortality [26]. Moreover, meta-analyses indicate that CRP is also a strong predictor, despite the consideration of conventional risk factors, including troponin levels, age, and comorbidities [27]. All these results support the idea that CRP is a universal biomarker with prognostic value in various groups of people and healthcare systems [28].

Limitations and future directions

This study has several limitations. First, it was conducted at a single tertiary care center, which may limit the generalizability of the findings. Second, the observational design precludes establishing a causal relationship between elevated CRP levels and adverse cardiovascular outcomes. Third, although major confounders such as age, sex, diabetes, hypertension, and smoking were adjusted for, other potential influences - such as medication use, socioeconomic status, and genetic factors - were not assessed and may have affected CRP levels or outcomes. While CRP was re-evaluated at 30 days to assess the short-term inflammatory response, longer-term serial measurements were not performed. Therefore, the sustained prognostic value of CRP beyond 30 days could not be determined in this study. Additionally, the relatively short follow-up period limits conclusions regarding long-term outcomes.

Future research should aim to validate these findings in larger, multicenter cohorts to enhance external validity. Longitudinal studies with extended follow-up durations are needed to evaluate the long-term prognostic significance of CRP levels after AMI. Moreover, randomized controlled trials investigating interventions that modulate CRP or systemic inflammation could provide insight into potential therapeutic implications. Finally, subgroup analyses involving patients with comorbidities, such as diabetes or chronic kidney disease, may help clarify the prognostic relevance of CRP across different clinical populations.

## Conclusions

High levels of CRP at admission are highly correlated with a high risk of 30-day MACE among patients with AMI. CRP is an objective and valid biomarker of early risk stratification, representing the severity of myocardial damage and systemic inflammation. The inclusion of CRP measurement in standard clinical evaluation can aid in the process of detecting high-risk patients who can be treated with special attention and intervention.
